# Polydimethylsiloxane tissue-mimicking phantoms with tunable optical properties

**DOI:** 10.1117/1.JBO.27.7.074706

**Published:** 2021-11-18

**Authors:** Aaron M. Goldfain, Paul Lemaillet, David W. Allen, Kimberly A. Briggman, Jeeseong Hwang

**Affiliations:** a National Institute of Standards and Technology, Sensor Science Division, Gaithersburg, Maryland, United States; b National Institute of Standards and Technology, Applied Physics Division, Boulder, Colorado, United States

**Keywords:** tissue-mimicking phantom, polydimethylsiloxane, integrating sphere, scattering coefficient spectrum, absorption coefficient spectrum

## Abstract

**Significance:**

The polymer, polydimethylsiloxane (PDMS), has been increasingly used to make tissue simulating phantoms due to its excellent processability, durability, flexibility, and limited tunability of optical, mechanical, and thermal properties. We report on a robust technique to fabricate PDMS-based tissue-mimicking phantoms where the broad range of scattering and absorption properties are independently adjustable in the visible- to near-infrared wavelength range from 500 to 850 nm. We also report on an analysis method to concisely quantify the phantoms’ broadband characteristics with four parameters.

**Aim:**

We report on techniques to manufacture and characterize solid tissue-mimicking phantoms of PDMS polymers. Tunability of the absorption ($\mu_{a}(\lambda )$) and reduced scattering coefficient spectra ($\mu_{s}^{\prime }(\lambda )$) in the wavelength range of 500 to 850 nm is demonstrated by adjusting the concentrations of light absorbing carbon black powder (CBP) and light scattering titanium dioxide powder (TDP) added into the PDMS base material.

**Approach:**

The $\mu_{a}(\lambda )$ and $\mu_{s}^{\prime }(\lambda )$ of the phantoms were obtained through measurements with a broadband integrating sphere system and by applying an inverse adding doubling algorithm. Analyses of $\mu_{a}(\lambda )$ and $\mu_{s}^{\prime }(\lambda )$ of the phantoms, by fitting them to linear and power law functions, respectively, demonstrate that independent control of $\mu_{a}(\lambda )$ and $\mu_{s}^{\prime }(\lambda )$ is possible by systematically varying the concentrations of CBP and TDP.

**Results:**

Our technique quantifies the phantoms with four simple fitting parameters enabling a concise tabulation of their broadband optical properties as well as comparisons to the optical properties of biological tissues. We demonstrate that, to a limited extent, the scattering properties of our phantoms mimic those of human tissues of various types. A possible way to overcome this limitation is demonstrated with phantoms that incorporate polystyrene microbead scatterers.

**Conclusions:**

Our manufacturing and analysis techniques may further promote the application of PDMS-based tissue-mimicking phantoms and may enable robust quality control and quality checks of the phantoms.

## 1 Introduction

Tissue-mimicking phantoms with well-defined wavelength-dependent absorption and scattering coefficients are essential for the development, calibration, and evaluation of optical medical devices designed to measure the broadband optical properties of tissues.^1^^,^^2^ The absorption and scattering coefficients of biological tissues are proportional to the number of photons absorbed or scattered per centimeter of tissue traversed. These coefficients range over several orders of magnitude and are not necessarily correlated with each other, so robust methods to independently control and characterize the wavelength-dependent absorption and scattering coefficients of tissue-mimicking phantoms are crucial.^1^

A variety of materials have been explored for making tissue-mimicking phantoms. Studies on liquid phantoms have demonstrated that the total attenuation or back reflection of light is adjustable by varying the concentrations of light absorbing India ink or dyes and light scattering intralipids.^3^^–^^5^ A more comprehensive study on solid phantoms has been reported by Pifferi et al.^6^ and Sekar et al.^7^ Pifferi et al.^6^ established a MEDPHOT (optical methods for medical diagnosis and monitoring of diseases) protocol to make epoxy-resin-based phantoms with various concentrations of titanium dioxide (${\mathrm{TiO}}_{2}$) powder and black toner powder included as scattering and absorbing agents, respectively. The MEDPHOT sample set has been used for round robin tests of various optical medical devices. Sekar et al.^7^ demonstrated a recipe to reproducibly fabricate phantoms with room-temperature-vulcanizing silicone that include various concentrations of black silicone pigment (Polycraft Black Silicone Pigment) for applications in the 600- to 1100-nm wavelength (λ) range, commonly used for human organ studies.

During the past decade, polydimethylsiloxane (PDMS) has been increasingly used to make phantoms that mimic the optical, physical, and thermal properties of biological tissues and to evaluate the performance of various optical biomedical devices.^8^^–^^15^ PDMS has broadly been used for the fabrication of a variety of microfluidic, optical, and bioassay components and devices due to its excellent processability, durability, flexibility, and limited tunability of its optical, mechanical, and thermal properties.^16^^–^^19^ Researchers have discovered that these properties are beneficial for manufacturing tissue-mimicking phantoms as well. The PDMS base material is fluidic at room temperature, but by adding crosslinking curing reagents (methylhydrogenated silica, dimethylvinylated silica or trimethylated silica), PDMS molecules slowly polymerize to turn the fluid into an elastomeric solid in a few hours to days, depending on the curing temperature. Its final viscoelastic mechanical property is adjustable by controlling the proportion of curing reagent, the curing temperature, and cure time,^20^^,^^21^ enabling the PDMS to mimic the Young’s modulus (≈0.1 to ≈1  MPa) of soft biological tissues.^22^ The initial fluid phase and slow solidification time allow for manufacturing custom structures by conformal replication of the shape of complex molds. The cured and properly stored PDMS solids are mechanically and chemically stable for at least several months,^9^ so they can be readily shared among laboratories for round-robin evaluations of optical medical devices. Most biological tissues (e.g., liver, breast, cartilage, kidney, and stomach) exhibit refractive indices between 1.4 and 1.5 at visible wavelengths,^23^ and a recent study demonstrated that the refractive index of cured PDMS is tunable from 1.405 to 1.445 by adjusting the curing temperature or the proportions of the base and curing agents.^21^ The optical transparency and no fluorescent background of PDMS in the visible and near-infrared wavelength range up to 1100 nm^24^ also allows the material’s scattering and absorption coefficients to be controlled by adding pigments, absorbers, and scatterers to mimic the optical properties of different types of biological tissues.^1^

Various types of light absorbers including India ink, nigrosin dye, and coffee have been used to tune the absorption coefficient of PDMS phantoms, and light scatterers including ${\mathrm{TiO}}_{2}$ and aluminum oxide particles have been used to adjust the scattering coefficient of phantoms.^15^^,^^25^^–^^28^ Ayers et al.^15^ showed that the absorption or scattering coefficient depends on the concentrations of absorbing or scattering additives, India ink or ${\mathrm{TiO}}_{2}$, dispersed in the PDMS base material. However, the demonstration was limited to two samples with two different concentration combinations of the India ink and ${\mathrm{TiO}}_{2}$ particles. Studies by Madsen et al.^3^ and Di Ninni et al.^4^ reported that tuning the concentration of light absorbing India ink to independently control the absorption coefficient is challenging because the ink particles also induce size-dependent light scattering. Also studies by Greening et al. demonstrated that the absorption and reduced scattering coefficients at six different wavelengths can be adjusted by controlling the concentration of nigrosin dye and ${\mathrm{TiO}}_{2}$ but also found that the reduced scattering coefficient at each wavelength is inversely proportional to the concentration of the light absorber, nigrosin.^28^

Here we report on a robust technique to fabricate PDMS-based tissue-mimicking phantoms where the broad range of scattering and absorption properties are independently adjustable in the visible to near-infrared wavelength range, from 500 to 850 nm. We also report on an analysis method to quantify the phantoms’ broadband optical properties with four fitting parameters. Details are described below.

In our phantoms, we use ${\mathrm{TiO}}_{2}$ powder (TDP, particle size of 0.3 to 1.0  μm, TI-602 Atlantic Equipment Engineers, Inc.) as a scattering additive and carbon black powder [CBP, particle size 1 to 2  μm, FE-603, Atlantic Equipment Engineers Inc.)] as an absorbing additive. CBP is a highly absorbing material and the concentration of CBP required to mimic the absorption coefficients of biological tissues is <0.01% (mass fraction) of the base material.^29^^–^^31^ Significantly, higher concentrations (e.g., 0.1% TDP to obtain $\mu_{s}^{\prime }\approx 10\;{\mathrm{cm}}^{-1}$ at 700 nm) of scattering additives are typically required to produce scattering coefficients similar to biological tissues,^32^ so light scattering by the CBP is expected to be insignificant. On the other hand, because TDP exhibits virtually no absorption in the visible to near-infrared wavelength region (extinction coefficient of TDP at λ>500  nm is $<1\times 10^{-6}$),^33^ absorption by TDP is expected to be insignificant. Therefore, in our recipe, adjusting the concentrations of the CBP and TDP additives in the PDMS base material allows for independent tuning of the absorption and scattering coefficients in a broad wavelength range, as demonstrated in Sec. 3.

We also report a method to analyze the wavelength-dependent absorption and reduced scattering coefficient spectra ($\mu_{a}(\lambda )$ and $\mu_{s}^{\prime }(\lambda )$) of phantoms that include CBP and TDP at various concentration combinations. Our method allows for concise and systematic comparisons of the broadband optical properties of the phantoms using two wavelength-dependent fitting functions with a total of only four fitting parameters. It has been reported that the absorption spectrum of well-dispersed CBP can be approximated by a linear equation in the wavelength range of 500 to 800 nm,^34^ therefore, the absorption coefficient spectra dominated by the CBP concentration are fitted by the following empirical linear fitting function:

$$\mu_{a}(\lambda )=c\lambda +d.$$ (1)

Here, by definition of the absorption coefficient, $d\approx \rho_{a}\sigma_{a}$ for light absorbing particles in a non-absorbing PDMS medium, where $\rho_{a}$ is the number density of the absorbing particles and $\sigma_{a}$ is the absorption cross section of a single absorber. The term cλ describes how $\mu_{a}(\lambda )$ varies with wavelength but is expected to be insensitive to particle number density, where c is an adjustable parameter. The reduced scattering coefficient spectra are fitted by the following empirical power law fitting function that has previously been used to fit the reduced scattering coefficient spectra of biological tissues:^1^

$$\mu_{s}^{\prime }(\lambda )=a{\left(\lambda /500\right)}^{-b}.$$ (2)

Here, by definition of the reduced scattering coefficient, $\mu_{s}^{\prime }=\mu_{s}(1-g)$ and $a\approx \rho_{s}\sigma_{s}(1-g)$ for light scattering particles in a non-scattering PDMS medium, where g is the scattering anisotropy parameter, $\rho_{s}$ is the number density of the scattering particles, and $\sigma_{s}$ is the size-dependent scattering cross section of a single scatterer. The factor ${\left(\lambda /500\right)}^{-b}$ accounts for the wavelength dependence of $\mu_{s}^{\prime }(\lambda )$. The exponent b mainly reflects how the particle scattering cross section varies with particle size and the wavelength and is expected to be independent of particle number density. The b value would be 4 in the Rayleigh regime (particle size ≪λ) and decreases as the particle size becomes comparable to or larger than λ. Thus if particles larger than the wavelength are also included, the b value is expected to decrease as the scattering contribution from Rayleigh particles becomes less significant.^1^ Our analysis quantifies the phantoms’ characteristics with the four parameters in these two fitting equations to enable a concise report of their broadband characteristics and comparisons to biological tissues.

## 2 Materials and Methods

### 2.1 PDMS Phantoms Including Both Light Scattering and Light Absorbing Particles

**Fig. 1:**
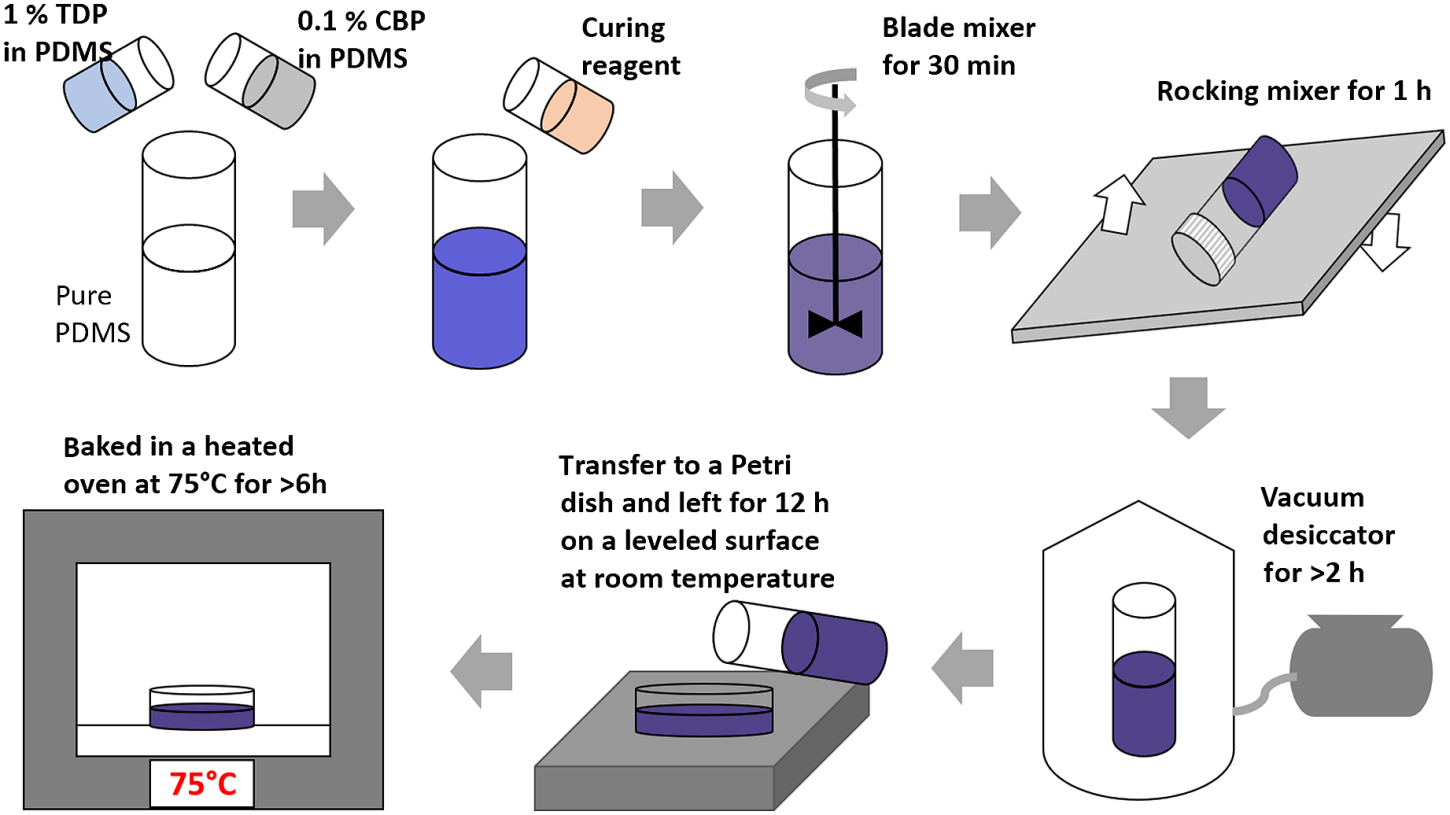
A step-by-step phantom fabrication procedure.

**Fig. 2:**
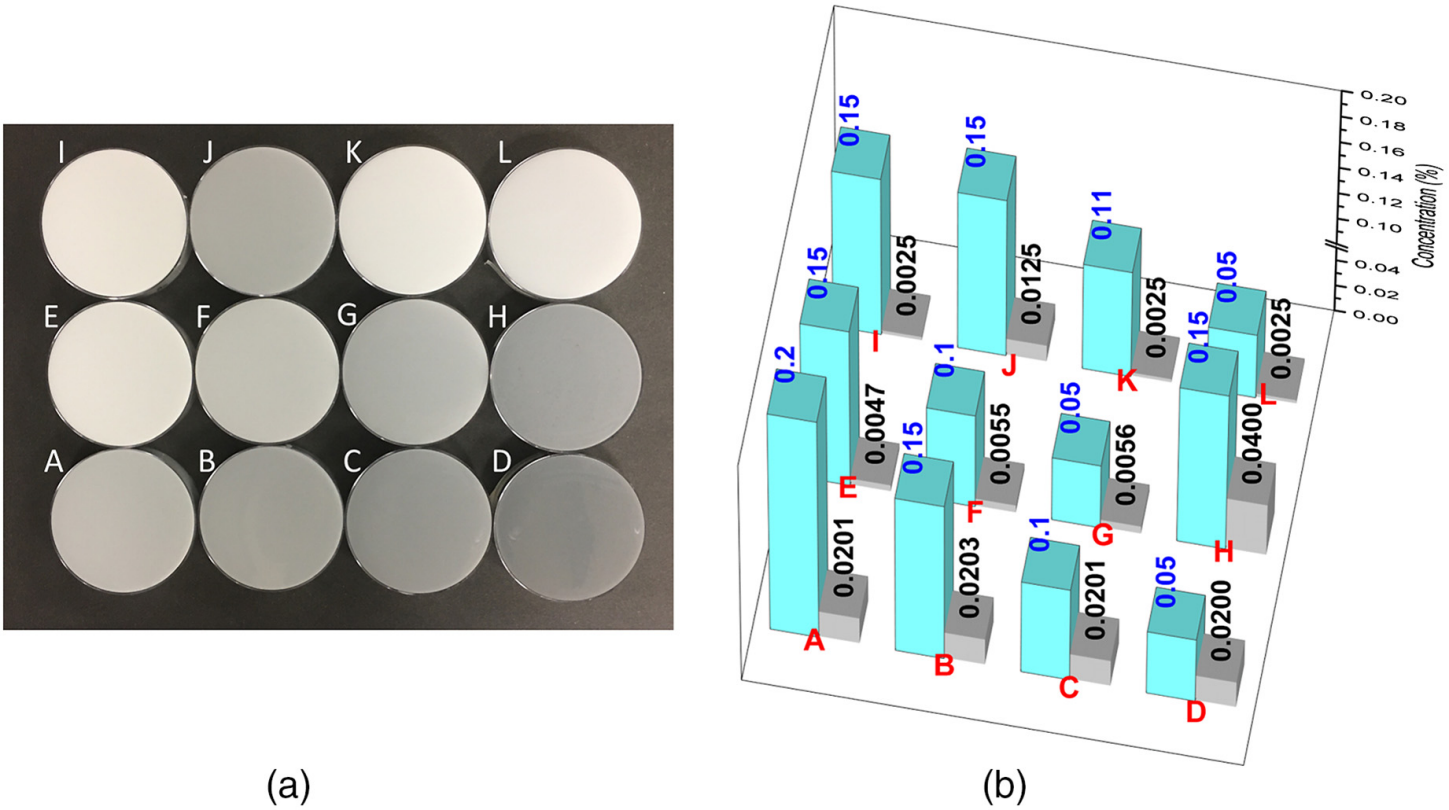
PDMS phantoms. (a) A set of PDMS samples with various concentration combinations of TDP and CBP. The diameter of each sample is 87.0 mm. (b) A 3D plot of the mass ratio percentages of TDP (cyan) and CBP (gray) with respect to PDMS, respectively, for the samples shown in (a). The numbers shown are actual mass ratio percentages of the additives in each sample.

PDMS-based phantoms with light scattering TDP and light absorbing CBP uniformly dispersed in PDMS (Sylgard 184 Elastomere, Corning) are prepared by the protocol described in detail elsewhere,^35^ and the protocol is illustrated in Fig. 1. In brief, two separate stock suspensions of PDMS, one with dispersed TDP (1% TDP in pure PDMS, w/w ratio) and the other with dispersed CBP (0.1% CBP in pure PDMS, mass ratio) are prepared. All weight measurements were done by a calibrated electronic counting scale balance with nominal measurement accuracy of 0.02 g (SC600, Extech Instruments). The proper amount of each stock suspension is further diluted into a container of pure PDMS to produce the final desired concentrations of TDP and CDP. Each stock suspension was prepared by adding a weighed amount of either CBP or TBP to pure PDMS in a 150-mL glass jar, mixing with a blade mixer for >30  min, then keeping the cap-closed jar on a rocking mixer overnight before use. For solid phantom fabrication, the curing reagent (mixture of methylhydrogenated silica, dimethylvinylated silica, and trimethylated silica, typically, 10% w/w to the PDMS-CBP-TDP mixture) is added into the suspension and thoroughly mixed with a blade mixer for 30 min followed by further mixing in a rocking mixer for another 1 h. As the curing process begins as soon as the curing agent is added to the PDMS suspension, the increase of the suspension’s viscosity during the rocking mixing process minimizes gravity-induced sedimentation due to decreased mobility of the particles. The sample mixture is then put in a vacuum desiccator for >2  h to remove trapped air bubbles and then gently poured into 87.0 mm diameter bacteriological Petri dishes (Nunc, Thermo Fisher). The poured sample is placed on a leveled surface and left for 12 h at room temperature for slow curing. The slow curing process prevents catastrophic phase separated aggregation of the additives, which occurs if the temperature is rapidly increased in an oven before the slow curing process. We did not see any noticeable sedimentation of the particles at the bottom of the cured samples. However, to check and mitigate potential measurement artifact due to any invisible micrometer scale heterogeneity across the sample thickness, we have performed reflectance measurements at two sample orientations, one with a sample in one orientation and the other with the same sample in a flipped orientation such that opposite face of the sample is facing the sphere. The two results agree with each other within the uncertainty bounds (data not shown). After curing at room temperature, the sample is then transferred into a 75°C oven and baked for >6  h for final curing. When cured, the sample is readily separable from the Petri dish molds. In phantom fabrication, we recognize the systematic fabrication protocol of the MEDPHOT phantoms described by Pifferi et al.^6^ The MEDPHOT set consists of total 32 phantoms with nominal $\mu_{a}$ = (0, 0.05, 0.10, 0.15, 0.20, 0.25, 0.30, and 0.35) ${\mathrm{cm}}^{-1}$ and nominal $\mu_{s}^{\prime }$ = (5, 10, 15, and 20) ${\mathrm{cm}}^{-1}$ at λ=800  nm, which have been widely used for a series of round robin tests. In this work, we fabricated phantoms with various TDP and CBP concentrations, targeting $\mu_{a}\approx 0$ to $3\;{\mathrm{cm}}^{-1}$ and $\mu_{s}^{\prime }\approx 2$ to $25\;{\mathrm{cm}}^{-1}$ at λ=800  nm, which sufficiently overlap with the ranges of the $\mu_{a}$ and $\mu_{s}^{\prime }$ of the MEDPHOT phantoms. Figure 2(a) shows a set of the cured PDMS phantoms in Petri dishes (87.0-mm diameter and about 2.5-mm thick) with concentrations of TDP and CBP in the range of 0.05% to 0.15% (w/w for TDP/PDMS) and 0.0025% to 0.04% (w/w for CBP/PDMS), respectively. Figure 2(b) shows a 3D plot of the mass ratio percentages of TDP and CBP with respect to PDMS.

A set of PDMS phantoms was also made with unfunctionalized polystyrene (PS) microbeads uniformly dispersed (1.00±0.05  μm diameter, PS04001, Bangs Laboratories, Inc.) at various concentrations. This set is comprised of the following three samples: (i) no TDP and 1.25% (w/w in PDMS) PS beads; (ii) 0.1% TDP and 0.625% PS beads; and (iii) 0.2% TDP and no PS beads. To disperse the PS beads in PDMS, the PS beads are first sedimented out of their stock aqueous buffer to the bottom of a centrifuge tube. The aqueous buffer was removed and the beads were dispersed in methanol and then added to uncured, pure PDMS or uncured, PDMS including TDP. The PDMS-bead solution is then placed onto a magnetic stirring plate for >3  h to allow the methanol to evaporate and then cured with the same method used for the PDMS-CBP-TDP phantoms.

### 2.2 Integrating Sphere System for the Measurement of Broadband Absorption and Scattering Coefficient Spectra

Our ultimate goal is to deploy our PDMS phantoms as measurement standards among multiple laboratories for a round robin test. The measurement goal of this work is to demonstrate that independent control of $\mu_{a}(\lambda )$ and $\mu_{s}^{\prime }(\lambda )$ is possible by systematically varying the concentrations of CBP and TDP in the same PDMS phantom. For these reasons, we focused on characterizing all of the optical properties of the PDMS phantoms, including any possible PDMS background. An integrating sphere system is used to measure the hemispherical reflectance and transmittance spectra of the phantoms and an inverse adding doubling (AD) algorithm is used to calculate the absorption and reduced scattering coefficients. The integrating sphere measurement system uses a single integrating sphere and is detailed elsewhere.^32^^,^^36^ In brief, broadband illumination (from 470 to 2400 nm) from a supercontinuum laser (SuperK EXB, NKT Photonics) is collimated and directed to the surface of the sample at the sample port of the integrating sphere (101002 UMBK-190, Gigahertz Optik GmbH). The illumination is a normally incident collimated Gaussian light beam through an aperture with 3.5 mm diameter clipping the beam at the full-width of the half-maximum of the Gaussian. To eliminate potential artifacts due to laser power fluctuations, about 2% of the illumination light is picked off and directed into a 50 mm diameter integrating sphere (IS200, Thorlabs Inc.). The light spectrum collected by this sphere is measured with a reference spectrophotometer (USB 2000, Ocean Optics, nominal resolution 8.5 nm) through a multi-mode optical fiber, then the spectrum is used for normalizing the signal spectrum from a main integrating sphere. The diffuse reflectance and transmittance (sphere rotated 180 deg) of the sample are detected by a signal spectrophotometer (USB 2000, Ocean Optics, nominal resolution 8.5 nm) connected to the main integrating sphere by a multi-mode optical fiber. Measurements of the phantom’s diffuse reflectance and transmittance are based on a substitution procedure that requires a comparison of the reflectance of the sample to the reflectance of a standard (NIST traceable standard reference material SRM 2044, which has nominally 99% reflectance over the wavelength range)^37^ and the transmittance of the standard to the transmittance with no sample. Calculations of $\mu_{a}(\lambda )$ and $\mu_{s}^{\prime }(\lambda )$ are done by the AD algorithm by Prahl,^38^ which solves the radiative transfer equation using a given $\mu_{s}^{\prime }$ and $\mu_{a}$ at each wavelength, with the thickness of the sample, the probe beam diameter, the particle size-dependent g, and the wavelength-dependent refractive index n(λ) of the PDMS as input parameters. The g value is set to 0.5, following Firbank and Delpy^39^ for wavelength-averaged submicrometer sized TDPs, and n(λ) for the surrounding PDMS medium and its uncertainty are taken from Niemeier and Rogers.^40^ The measurement results may depend on the g value and the measurement modality. Therefore, the same g value must be used for a round robin test of the phantoms by the same type of measurement instruments, integrating sphere systems in different laboratories. An iterative inversion procedure uses the AD algorithm to compare the measured reflectance and transmittance values with simulated results to determine the final $\mu_{a}$ and $\mu_{s}^{\prime }$, as well as the total uncertainty budget at each wavelength.^32^ The uncertainty budget accounts for the variation among five repeated measurements from one sample location as well as contributions due to uncertainties in the input parameters of sample thickness, the reflectance of the reference standard, and the refractive index of PDMS. We used a fixed beam diameter because the beam is illuminated through an aperture of fixed diameter.

## 3 Results and Discussion

**Fig. 3:**
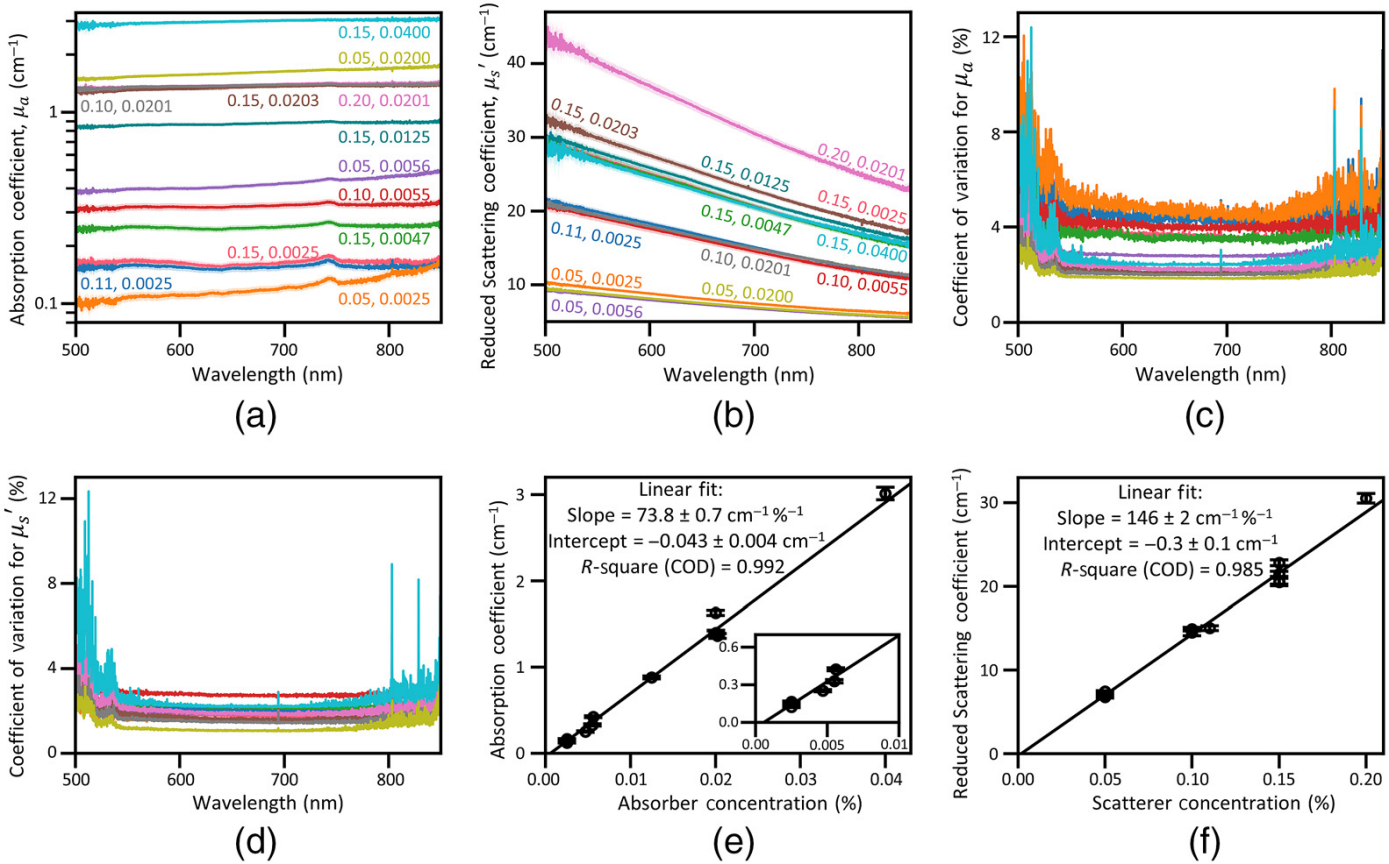
Optical properties of the phantoms. (a) Absorption and (b) scattering coefficients of the phantoms with both TDP and CBP at various concentration combinations across the wavelength range of 500 to 850 nm. The numbers labeling each curve are the concentrations (% w/w) of TDP and CBP, respectively. (c), (d) The coefficient of variation of the absorption and scattering coefficients of each sample. The curve for each sample is plotted using the same color scheme as in (a) and (b). (e) Absorption and (f) reduced scattering coefficients at 700 nm versus the concentration of CBP and TDP, respectively, demonstrating that both coefficients depend linearly on the concentration of the corresponding additive. Note that high CODs confirm excellent linear fit for both cases. Other details of the fitting results are listed as well. The inset of (e) shows the absorption coefficient at low absorber concentration. In (a), (b), (e), and (f), the plotted uncertainties [shaded areas in (a) and (b) and the bars in (e) and (f)] for the coverage factor k=1 are calculated as described in Sec. 2.

Figures 3(a) and 3(b) show the wavelength dependence of $\mu_{a}(\lambda )$ and $\mu_{s}^{\prime }(\lambda )$ of phantoms that include both TDP and CBP at various concentration combinations. Figures 3(c) and 3(d) show the wavelength dependence of the coefficient of variation of $\mu_{a}(\lambda )$ and $\mu_{s}^{\prime }(\lambda )$. The coefficient of variation remains below 6% at most wavelengths but increases near the edges of the measured spectral region where our integrating sphere measurement setup has a reduced signal-to-noise ratio. Each absorption coefficient spectrum $\mu_{a}(\lambda )$ in Fig. 3(a) is almost flat over the entire wavelength range except the one (orange colored) at the lowest concentrations of CBP (0.05%) and TDP (0.0025%), which shows increasing absorption coefficients as the wavelength increases. This behavior is likely due to a systematic measurement error induced when measuring $\mu_{a}(\lambda )$ and $\mu_{s}^{\prime }(\lambda )$ of finite thickness samples with the integrating sphere system, resulting in collimated transmission due to reduced scattering events.^41^^,^^42^ The small peak observed near 740 nm is due to weak optical absorption by PDMS, likely a vibrational overtone of a methyl group ($─{\mathrm{CH}}_{3}$) stretch absorption.^24^^,^^43^ For the reduced scattering coefficient $\mu_{s}^{\prime }(\lambda )$, Fig. 3(b) shows significant changes with wavelength. The increase of $\mu_{a}(\lambda )$ with wavelength is not correlated with the TDP concentration, indicating that the absorption coefficients at all wavelengths depend only on the concentration of CBP not TDP. As an example, Fig. 3(e) presents $\mu_{a}$ at 700 nm, which increases linearly with CBP concentration. We confirm that this linear relation holds for all wavelengths, finding that the coefficient of determination (COD, R-square) is always >0.975 (data at other wavelengths not shown). The inset of Fig. 3(e) highlights the absorption coefficients at low absorber concentrations at 700 nm, where the absorption coefficient is similar to that of tissues in the near infrared spectral region, 650 to 1100 nm.^1^

**Fig. 4:**
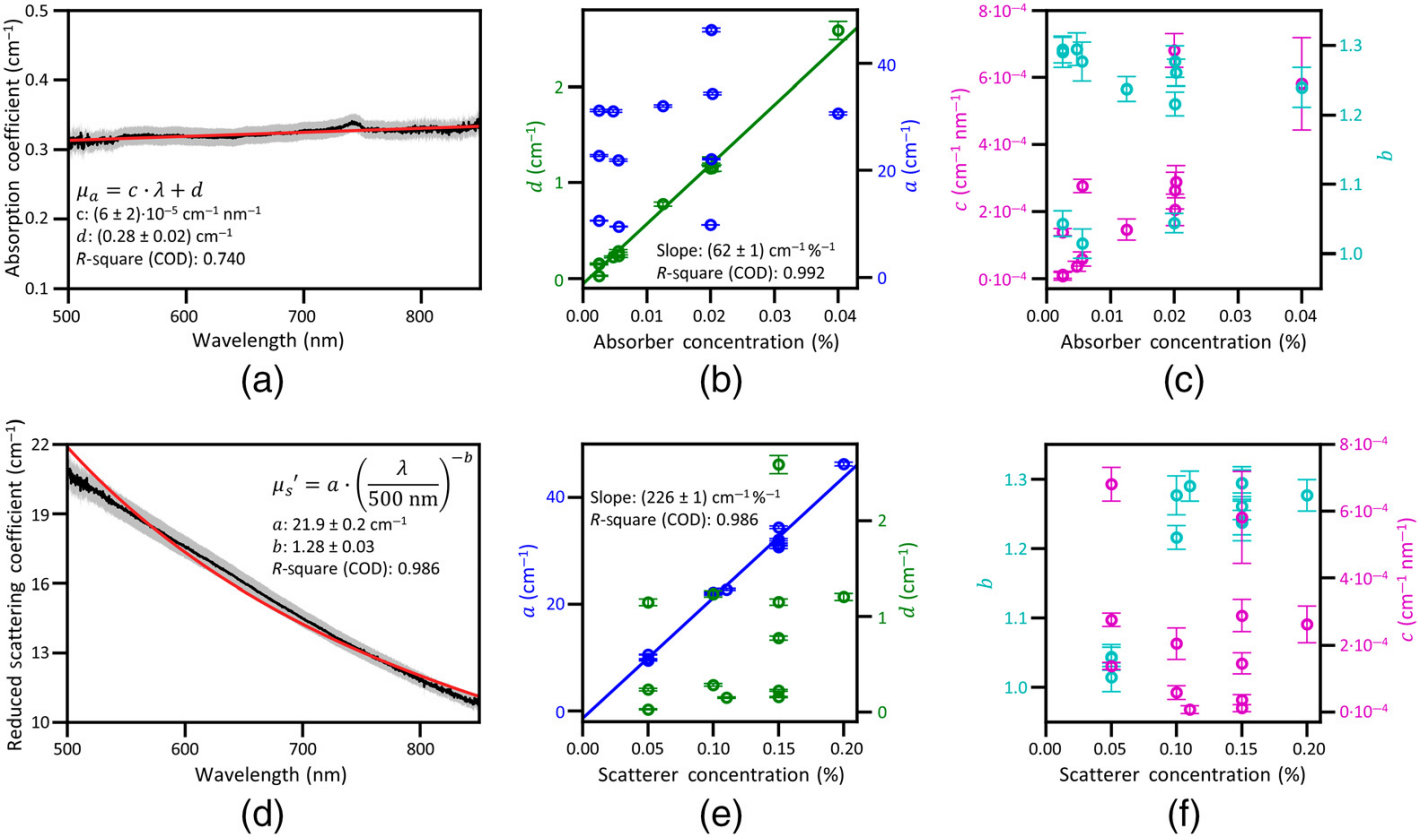
Analyses of the absorption and reduced scattering spectra. (a) An absorption coefficient spectrum $\mu_{a}(\lambda )$ for a phantom containing scattering TDP and absorbing CBP with concentrations of 0.1% and 0.0055% (w/w), respectively, and fit to a wavelength-dependent linear function, $\mu_{a}(\lambda )=c\cdot \lambda +d$. The gray area represents an uncertainty bound (k=1) of experimental data. (b), (c) Plots of the fitted parameters (a,b,c,d) versus CBP concentration. (d) A reduced scattering coefficient $\mu_{s}^{\prime }(\lambda )$ for the same sample as in (a) and fit to a wavelength-dependent power law function, $\mu_{s}^{\prime }(\lambda )=\;a{\left(\lambda /500\right)}^{-b}$. The gray area represents an uncertainty bound (k=1) of the experimental data. (e), (f) Fit parameters (a, b, c, and d) versus TDP concentration (e) and (f). In (b) and (e), linear fits to the parameters d and a, respectively, are shown along with slopes and CODs of the linear fits.

Figure 3(b) clearly shows that the reduced scattering coefficients for all samples decay as the wavelength increases. The spectral data curves move up as TDP concentration increases, but the increase is not strongly correlated with CBP concentration, indicating that the reduced scattering coefficients at all wavelengths depend only on the concentration of TDP not CBP. As an example, Fig. 3(f) presents $\mu_{s}^{\prime }$ at 700 nm, which linearly increases with TDP concentration. We confirm that this linear relation holds for all wavelengths, finding that the COD is always >0.96 (data at other wavelengths not shown). The spread in the $\mu_{s}^{\prime }(\lambda )$ curves for all samples with the same TDP concentration (0.15%) is within the uncertainty bounds except for one with a CBP concentration of (0.0203%, in brown in Fig. 3). The percentages of the CBP and TDP in the final forms of phantoms are calculated values from the dilution. This curve shows a slightly higher values likely due to locally concentrated TDP at the laser illuminated area due to imperfect homogenization. The slopes of the reduced scattering coefficient spectra exhibit clear decay as the wavelength increases. The wavelength-dependent slopes at various TDP concentrations are discussed in depth later in this section [see Fig. 4(f)]. On the other hand, the absorption coefficient spectra show no obvious wavelength dependence and have small slopes.

To further quantify the wavelength and concentration dependencies described above, we fit the $\mu_{a}(\lambda )$ and $\mu_{s}^{\prime }(\lambda )$ spectral data of each sample to a linear equation [Eq. (1)] and a power law equation [Eq. (2)], respectively. Figures 4(a) and 4(d) show typical examples of the $\mu_{a}(\lambda )$ and $\mu_{s}^{\prime }(\lambda )$ spectra of a phantom including both TDP and CBP at concentrations of 0.1% and 0.0055%, respectively. These plots demonstrate that the experimentally measured coefficients are well described by the fit functions within the measurement uncertainty. As shown in Figs. 4(a) and 4(d), nearly all data points (in black) are consistent with the fit (in red) within an uncertainty bound corresponding to a coverage factor k=1 (plotted in gray) for all phantoms (data not shown).

Analysis with these two fitting functions allows for concise tabulation of the phantoms’ optical characteristics using the four parameters (a, b, c, and d) in the fitting equations. The fit parameters (a, b, c, and d) are plotted against either CBP or TDP concentration in Figs. 4(b), 4(c), 4(e), and 4(f) for all phantoms. In Fig. 4(b), the d values (green) quantify the increase of $\mu_{a}(\lambda )$ with CBP concentration. An overlaid linear fit (green line) in the same plot shows the expected linear correlation between d and the CBP concentration with a COD of 0.992. The linear relation suggests that the total light absorption is determined predominantly by absorber particles since $\mu_{a}=\rho_{a}\sigma_{a}$. In the same plot, the a values (blue), which quantify the increase of $\mu_{s}^{\prime }(\lambda )$ with CBP concentration, do not correlate with TDP concentration. Thus $\mu_{a}(\lambda )$ depends only on the concentration of light absorbing CBP but $\mu_{s}^{\prime }(\lambda )$ does not.

To investigate how the spectral shape changes with absorber concentration, the c and b values that quantify the slope and the exponent in Eqs. (1) and (2), respectively, are plotted against CBP concentration in Fig. 4(c). This plot shows that the parameter b is not correlated with CBP concentration. The fit parameter c is somewhat correlated with CBP concentration, but because $\mu_{a}$ varies only weakly with wavelength, all values of c are small. Thus, even though c weakly depends on the absorber concentration, the phantoms’ $\mu_{a}(\lambda )$ are not strongly affected by this dependency. Because $\mu_{a}(\lambda )$ is not strongly affected by how c and b [the wavelength-shaping modification factors in Eqs. (1) and (2)] vary with CBP concentration, these results suggest the wavelength dependency of $\mu_{a}(\lambda )$ is mainly defined by the intrinsic optical properties of the CBPs not their concentration.

In Fig. 4(e), the a values (blue) quantify the increase of $\mu_{s}^{\prime }(\lambda )$ with TDP concentration. An overlaid linear fit (blue line) in the same plot shows a clear linear relation between a and TDP concentration with a COD of 0.986. Again, the linear relation suggests that the total light scattering is determined predominantly by the scattering particles TDP since $\mu_{s}=\rho_{s}\sigma_{s}$. In the same plot, the d values (green), which quantify the increase of $\mu_{a}(\lambda )$ with CBP concentration, do not correlate with TDP concentration. Thus $\mu_{s}^{\prime }(\lambda )$ depends only on the concentration of light scattering TDP but $\mu_{a}(\lambda )$ does not.

To investigate how the spectral shape changes with scatterer concentration, the b and c values that quantify the exponent and the slope in Eqs. (1) and (2), respectively, are plotted against TDP concentration in Fig. 4(f). This plot shows that the parameter c does not correlate with TDP concentration, but the parameter b weakly correlates with TDP concentration. We note that the b values may be divided into two groups: one with b<1.1 at the TDP concentration of 0.05% and the other with b>1.1 at higher (≥0.10%) TDP concentrations, but they are distributed within the small range between 1.02 and 1.28. Together, these results indicate that the spectral shape of $\mu_{s}^{\prime }(\lambda )$ changes slightly as TDP concentration increases, but the dominant factor affecting $\mu_{s}^{\prime }(\lambda )$ is the parameter a. Thus similar to $\mu_{a}(\lambda )$, because $\mu_{s}^{\prime }(\lambda )$ is not strongly affected by how b and c vary with TDP concentration, these results suggest that the wavelength dependency of $\mu_{s}^{\prime }(\lambda )$ is mainly defined by the intrinsic optical properties of the TDPs not their concentration.

To summarize, the correlation between d and CBP concentration in Fig. 4(b) shows that CBP concentration is the primary factor that determines the absorption coefficient magnitude at all wavelengths. Similarly, the correlation between a and TDP concentration in Fig. 4(e) shows that TDP concentration is the primary factor that determines the reduced scattering coefficient magnitude at all wavelengths. The spectral shapes of $\mu_{a}(\lambda )$ and $\mu_{s}^{\prime }(\lambda )$ vary slightly with CBP and TDP concentration, respectively, but these shape variations are subtle and have little effect on the overall absorption and scattering coefficients. The weak correlation of the parameters b and c with CBP and TBP concentration, respectively, seen in Figs. 4(c) and 4(e), results from these variations in spectral shape. The physical reasons underlying the slight variations in the spectral shapes will be the subject of future work. Possible reasons include the emergence of multiple scattering effects, clumping of particles at higher particle concentrations, or a small amount of light absorption by the PDMS media.

To evaluate the optical properties of our phantoms toward practical uses, we compare their optical properties to those of human tissues. For the comparison, we use the scattering characteristics (within the 400 to 1300 nm wavelength range) of various types of human tissues summarized in a review paper by Jacques.^1^ We focus only on the comparison of the scattering spectra because the absorption spectra of most human tissues contain localized light absorbing molecules with unique absorption features over specific wavelength regions. Fabrication of PDMS phantoms mimicking such unique absorption spectral features is beyond the scope of this study. The wavelength dependence of $\mu_{s}^{\prime }(\lambda )$ of our phantoms resembles that of various types of human tissues, and both can be fit by Eq. (2).

**Fig. 5:**
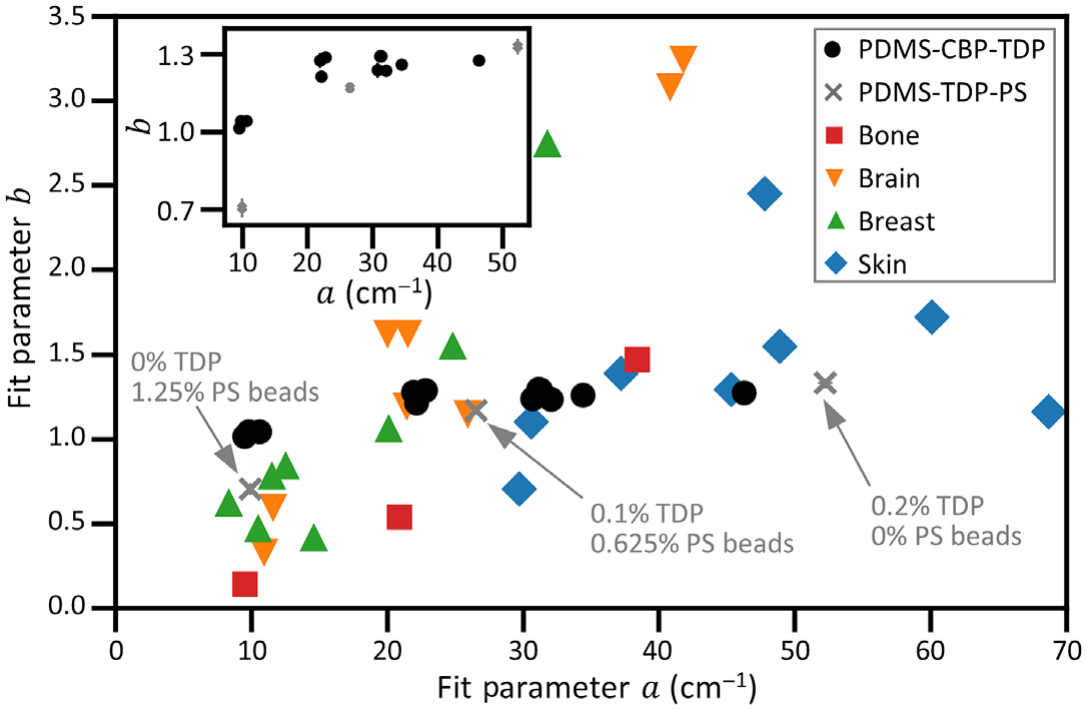
Comparison of the optical properties of phantoms versus human tissue. A plot of b versus a for human tissues and PDMS phantoms, where the parameters a and b are defined in Eq. (2). The a and b parameters for tissues are taken from Jacques (Ref. 1). The inset shows the same parameters for PDMS phantoms with CBP and TDP (round dots) and three PDMS phantoms with varying concentrations of TDP and PS beads (“x” marks). The vertical bars represent uncertainty bounds (k=1), although in most cases the plotted uncertainty is smaller than the point size.

Figure 5 shows plots of b versus a for both human tissues and our phantoms, where the parameters a and b are defined in Eq. (2). The a and b parameters for tissues are taken from Jacques.^1^ We note that the distribution of the phantoms’ a values covers a broad range from 10 to $45\;{\mathrm{cm}}^{-1}$, mimicking the a values for most tissues except for a few reported values for skin, which show very high a values. As a depends mainly on the individual particle scattering cross sections and their number density, a can be readily tuned with these two manufacturing parameters. Meanwhile, Fig. 5 shows that the distribution of b values for the PDMS-CBP-TDP phantoms is limited between 1 and 1.2, which is much narrower than that of the b values for tissues. Recall that the parameter b is the exponent of the power law fitting function, which determines the wavelength dependency of $\mu_{s}^{\prime }(\lambda )$ and is primarily sensitive to particle size. Some tissues show high b values approaching 4, suggesting that Rayleigh scattering is more important in these tissues. To mimic these tissues, additional scattering particles that are smaller than the wavelength of the illumination could be added to the phantoms in the future work.

Some tissues show b values smaller than those of our phantoms, with some b values being even smaller than 1. These low b values suggest that additional particles with sizes larger than the wavelength of the light (and larger than those used in this study) could be added to the PDMS phantoms to further decrease b and mimic those tissues. To demonstrate that adding such particles decreases the relative amount of Rayleigh scattering and lowers b, we manufactured and characterized three PDMS phantoms with different concentration ratios of 1-μm-diameter PS beads to TDP. Note that the mean diameter of these PS beads is the same as the upper limit of the TDP size distribution, but the diameters are more narrowly distributed. The addition of such larger diameter 1-μm PS beads and the lower refractive index of the PS bead (≈1.58) as opposed to that of TDPs (≈2.87) helps to decrease the contribution by Rayleigh scattering off the particles. The three phantoms tested include the following particles: (i) no TDP and 1.25% (w/w in PDMS) PS beads; (ii) 0.1% TDP and 0.625% PS beads; and (iii) 0.2% TDP and no PS beads. We fit the $\mu_{s}^{\prime }(\lambda )$ spectral data of these samples to a power law equation [Eq. (2)] to obtain a and b values. The $\mu_{s}^{\prime }(\lambda )$ data from phantoms included with PS beads exhibited a slight oscillatory behavior, barely observable over the uncertainty. The oscillation is small enough to allow for a good fit to a power law equation with a high COD, 0.995 (data not shown). As expected, the scattering by sample (i) shows less Rayleigh scattering, as characterized by a substantially decreased b value (data point “x” toward the lower left corner in Fig. 5 and the inset). The inset of Fig. 5 shows a plot of a versus b for phantoms (i), (ii), and (iii) (‘x’ marks with vertical and horizontal error bars overlaid) to compare them with the parameters of the PDMS-CBP-TBD phantoms (round dots) over a reduced (a,b) parameter space. Uncertainties (k=1) of the PDMS phantom data points are included in both Fig. 5 main plot and the inset, confirming that the change in b is significant across the PS phantom set.

Repeatability to manufacture phantoms of the same optical properties and reproducibility of the measurement results through multi-laboratory tests are essential to ensure long-term and interlaboratory usage of the phantoms. The multi-laboratory measurement requires high-fidelity instruments that are validated for their performance. For these reasons, we performed such tests in a limited capacity with a subset of our phantoms and have reported the results elsewhere.^32^ For Ref. 32, we manufactured two sets of samples of the same optical properties, following the same recipe, and measured each set at two different laboratories and confirmed the results from two laboratories agree each other. Further tests involving more laboratories are in our future plan.

## 4 Conclusion

We report on techniques to manufacture and characterize PDMS phantoms mimicking optical properties of human tissues with a broad range of absorption and reduced scattering coefficients. Controlling the concentrations of light absorbing CBP and light scattering TDP dispersed in the PDMS phantom allows for independent tuning of $\mu_{a}(\lambda )$ and $\mu_{s}^{\prime }(\lambda )$ from 0.1 to $3\;{\mathrm{cm}}^{-1}$ and from 1 to $47\;{\mathrm{cm}}^{-1}$, respectively, in the wavelength range of 500 to 850 nm. We measure the phantoms’ $\mu_{a}(\lambda )$ and $\mu_{s}^{\prime }(\lambda )$ spectra with a broadband integrating sphere system and fit the spectra with a linear or power law equation for $\mu_{a}(\lambda )$ or $\mu_{s}^{\prime }(\lambda )$, respectively. Nearly, all data points of all phantoms are consistent with the fit within the k=1 uncertainty bound, demonstrating that the experimentally measured coefficients are well described by these fit functions. Our analysis using empirical linear or power law fitting functions concisely quantifies the optical properties of the phantoms using a total of four parameters for these two fitting equations. The analysis and fitting parameters confirm that our fabrication technique allows for independent control of the phantom’s $\mu_{a}(\lambda )$ or $\mu_{s}^{\prime }(\lambda )$ by separately adjusting the concentration of CBP or TDP, respectively. The PDMS phantoms mimic the magnitudes of the absorption and reduced scattering coefficients of various types of human tissues but only reproduce the spectral shapes found in human tissues to a limited extent with a given scattering anisotropy factor g=0.5. The addition of PS particles that are larger than the wavelength of the illumination reduces Rayleigh scattering signatures suggesting that the spectral shape of the reduced scattering coefficient may be tuned to mimic various types of human tissues to a fuller extent. Our manufacturing and analysis techniques may further promote the application PDMS-based tissue-mimicking phantoms and may enable robust quality control and testing of the phantoms.

## Code, Data, and Materials Availability

Data used in this manuscript can be made available by a reasonable request to the corresponding author. Materials used in this manuscript can be made availabe through a materials transfer agreement.
